# Late presentation of cutaneous larva migrans: a case report

**DOI:** 10.4076/1757-1626-2-7553

**Published:** 2009-08-12

**Authors:** Michael Archer

**Affiliations:** Grimston Medical CentreCongham Road, Grimston, King's Lynn, Norfolk, PE32 1ANUK

## Abstract

**Introduction:**

Cutaneous larva migrans is caused by infection with hookworm larvae in tropical and sub-tropical areas. A history of recent travel to the tropics is usually elicited.

**Case presentation:**

A case of cutaneous larva migrans is described in which symptoms did not appear until five months after travel to Tanzania.

**Conclusion:**

Although the lesion of cutaneous larva migrans may appear immediately, the larvae may lie dormant for many months and presentation may therefore occur a long time after any foreign travel.

## Case presentation

**Figure 1.**
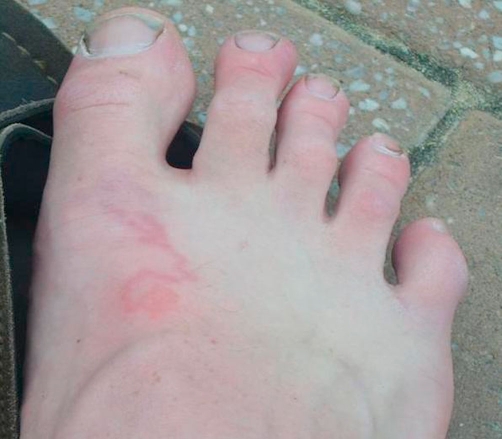
Photograph of right foot taken on date of presentation showing typical serpiginous lesion of cutaneous larva migrans.

An 18-year-old white British man presented with a three-day history of an intensely itchy eruption on the dorsum of his right foot. His symptoms started immediately after a long-haul flight from Britain to Australia. Five months previously he had travelled to Tanzania, where he walked barefoot on beaches. Examination revealed a typical serpiginous lesion (Figure 1) and a diagnosis of cutaneous larva migrans was made on clinical grounds. Treatment with oral mebendazole cured both the lesion and the itching within a week.

Cutaneous larva migrans is caused by infection with hookworm larvae in tropical and sub-tropical areas; a history of foreign travel and of walking barefoot on sandy soil or beaches can often be obtained [1]. The diagnosis is usually made on the basis of the typical appearance of the lesion, intense itching and history of foreign travel. The lesion is self-limiting, usually subsiding within 4-8 weeks. Treatment with anti-helminthic agents is recommended to shorten the course of the disease and reduce itching [2].

Although the lesion of cutaneous larva migrans may appear almost immediately, this case illustrates the fact that the larvae may lie dormant for many months after infection. Presentation may therefore occur a long time after travel to the tropics. It is possible that a subsequent long-haul flight was the precipitant that awakened the larva from its dormant stage in this case.
